# Supersonic shearwave elastography in the assessment of liver fibrosis for postoperative patients with biliary atresia

**DOI:** 10.1038/srep31057

**Published:** 2016-08-11

**Authors:** Shuling Chen, Bing Liao, Zhihai Zhong, Yanling Zheng, Baoxian Liu, Quanyuan Shan, Xiaoyan Xie, Luyao Zhou

**Affiliations:** 1 Department of Medical Ultrasonics, Institute of Diagnostic and Interventional Ultrasound , The First Affiliated Hospital of Sun Yat-sen University, No.58 Zhongshan Road 2, Guangzhou, 510080, P.R. China; 2 Department of pathology , The First Affiliated Hospital of Sun Yat-sen University, No.58 Zhongshan Road 2, Guangzhou, 510080, P.R. China; 3 Department of pediatric surgery, The First Affiliated Hospital of Sun Yat-sen University, Sun Yat-sen University, Guangzhou 510080, P. R. China

## Abstract

To explore an effective noninvasive tool for monitoring liver fibrosis of children with biliary atresia (BA) is important but evidences are limited. This study is to investigate the predictive accuracy of supersonic shearwave elastography (SSWE) in liver fibrosis for postoperative patients with BA and to compare it with aspartate aminotransferase to platelet ratio index (APRI) and fibrosis-4 (FIB-4). 24 patients with BA received SSWE and laboratory tests before scheduled for liver biopsy. Spearman rank coefficient and receiver operating characteristic (ROC) were used to analyze data. Metavir scores were F0 in 3, F1 in 2, F2 in 4, F3 in 7 and F4 in 8 patients. FIB-4 failed to correlate with fibrosis stage. The areas under the ROC curves of SSWE, APRI and their combination were 0.79, 0.65 and 0.78 for significant fibrosis, 0.81, 0.64 and 0.76 for advanced fibrosis, 0.82, 0.56 and 0.84 for cirrhosis. SSWE values at biopsy was correlated with platelet count (r = −0.426, *P* = 0.038), serum albumin (r = −0.670, *P* < 0.001), total bilirubin (r = 0.419, *P* = 0.041) and direct bilirubin levels (r = 0.518, *P = *0.010) measured at 6 months after liver biopsy. Our results indicate that SSWE is a more promising tool to assess liver fibrosis than APRI and FIB-4 in children with BA.

Biliary atresia (BA), characterized by progressive fibro-obliteration of the bile ducts, is a common infantile cholestatic disease with high morbidity and mortality^1^. Kasai portoenterostomy (KPE) remains the initial strategy to restore bile flow^2^. However, despite of timely KPE, the progressive fibrosis develops in almost all patients^3^, which results in liver cirrhosis and requires subsequent liver transplantation (LT)^4^. The degree of liver fibrosis after KPE contributes to be the main prognostic factors for the survival of patients^5^. Therefore, close monitoring of fibrosis is critical for identifying high-risk patients and referring them to LT.

Liver biopsy is the criterion standard for evaluating liver fibrosis. However, as an invasive procedure, it has many limitations including complications, discomfort, inter-observer variations and sampling errors^6^,^7^. Given this situation, various non-invasive methods have rapidly emerged as alternatives to liver biopsy, such as quantitative elastography and serum fibrosis biomarkers.

Several quantitative elastography technologies such as transient elastography (TE), acoustic radiation force impulse (ARFI), and supersonic shearwave elastography (SSWE), have been used to evaluate liver fibrosis in pediatric patients and showed good correlation (ρ = 0.53–0.63) between liver fibrosis and elastographic value^8^,^9^,^10^,^11^. However, TE has many pitfalls such as the inability to choose different locations for the region of interest (ROI) and to avoid other structures such as liver vessels and bile ducts^12^, and has also been reported to have more technical failures in young children^13^,^14^. APRI, also named as point shear wave elastography, has the limitation in being unable to provide a real-time quantitative map of liver tissue stiffness^15^. SSWE, a newly developed elastography technology, has three advantages over TE. First, SSWE is integrated into a conventional diagnostic ultrasound (US) system and therefore can make use of real-time gray scale mode imaging for the assessment of morphologic changes or avoiding big vessels^16^. Second, improved separation of fibrosis stages due to the use of shear waves with greater bandwidths has improved its discriminative power in staging fibrosis^17^. Third, SSWE enables a real-time stiffnessanalysis with a real-time map of the elasticity in a region^18^, which showed benefit over TE and also ARFI. Previous adult studies have showed that it was a promising diagnostic tool in predicting cirrhosis^19^,^20^,^21^. However, to the best of our knowledge, there were very few studies regarding the performance of SSWE in liver fibrosis for children until now^15^,^22^. The aspartate aminotransferase (AST) to platelet (PLT) ratio index (APRI) and fibrosis-4 (FIB-4) score calculated from age, AST, alanine amino-transferase (ALT) and PLT, are both, inexpensive, easily available and non-invasive indices. They have been reported to be a surrogate marker of fibrosis and cirrhosis in adults with hepatitis C, hepatitis B, alcoholic hepatitis and nonalcoholic fatty liver^23^,^24^,^25^. However, their diagnostic performance for liver fibrosis in pediatric patients with different liver diseases including BA have exhibited variations among different studies (area under the receiver operating characteristic curves (AUROCs): 0.54–0.97)^26^,^27^,^28^.

This study aimed at evaluating the performance of SSWE to predict fibrosis stage in postoperative Chinese children with BA by comparing it with APRI and FIB-4, and to preliminarily investigate the correlation of SSWE with biochemical parameters of liver function at 6 months after liver biopsy.

## Materials and Methods

### Patients

This retrospective study based on the prospectively collected data was approved by the ethics committee of the First Affiliated Hospital of Sun Yat-sen University, and written informed parental consent was obtained from each patient. Our study was carried out in accordance with the approved guidelines. Between August 2012 and November 2015, 24 patients with BA followed at the First Affiliated Hospital of Sun Yat-sen University and scheduled for a liver biopsy were prospectively enrolled. All these patients underwent KPE before enrollment. SSWE examination and serum biochemical tests were offered to all the included patients almost in the same day or within 1 week of liver biopsy. Exclusion criteria were as follows: (1) failure of SSWE examination; (2) recent other diseases such as acute febrile illnesses or skin rashes, which could affect biochemical parameters (i.e. AST levels and PLT counts) other than BA; (3) co-infection with hepatitis B, hepatitis C, hepatitis D or HIV; (4) inadequate liver biopsies defined as having fewer than six portal tracts under the microscope; (5) no follow-up data. No patients were excluded in this study.

### Examination Protocols

#### Clinical and laboratory test

Demographic data including age and sex were recorded at the time of liver biopsy. During follow-up, liver related events such as prolonged jaundice, ascites, gastrointestinal bleeding, hepatic encephalopathy, liver transplantation and death were also recorded. Laboratory examinations were collected venously as part of routine clinical care throughout the follow-up. Data including AST, ALT, serum albumin (ALB), serum total bilirubin (TBIL), serum direct bilirubin (DBIL), gamma-glutamyl transpeptidase (GGT), alkaline phosphatase (ALP), and PLT count were obtained. Laboratory data obtained within 1 week of liver biopsy and at 6 months after liver biopsy were used for analysis in this study. The diagnosis of gastrointestinal bleeding was confirmed by endoscopic finding that bleeding developed from the varicose veins in the distal esophagus or gastric fundus^29^. Ascites was diagnosed when fluid was found in peritoneal cavity by imaging tests such as ultrasonography or computed tomography^30^. Jaundice (serum bilirubin ≥ 5 mg/dl [85 mmol/l]) was defined based on the guidelines of Asian Pacific Association for the study of liver^31^. APRI and FIB-4 were calculated according to the following formulas: APRI = (AST/upper limit of normal AST × 100)/ Platelet Count [10^9^/L], FIB-4 = (Age [years] × AST [U/L])/ (Platelets [10^9^/L] × (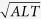 [U/L])). The upper normal range of AST were 37 IU/L.

#### SWE examination

An AixPlorer scanner (Supersonic, Paris, France) incorporating a SC6-1curvilinear transducer (1–6 MHz) was used to perform the SSWE examinations. The infants were remained still by sedation or by holding their breath. Segments V or VI were selected as the target areas for measurement. All SSWE examinations were performed by a single sonographer (Luyao Zhou, 6 years of experience for US and 3 years of experience for elastography, respectively) with an intercostal or a subcostal transducer position. If possible, SSWE was performed during a short breath hold (3 seconds). Otherwise, the acquisitions were made during one normal, gentle breathing cycle. The transducer was kept perpendicular to the skin and no press was given during the SSWE examination. Three measurements were made and recorded at the same segment for every infant.

**Figure 1:**
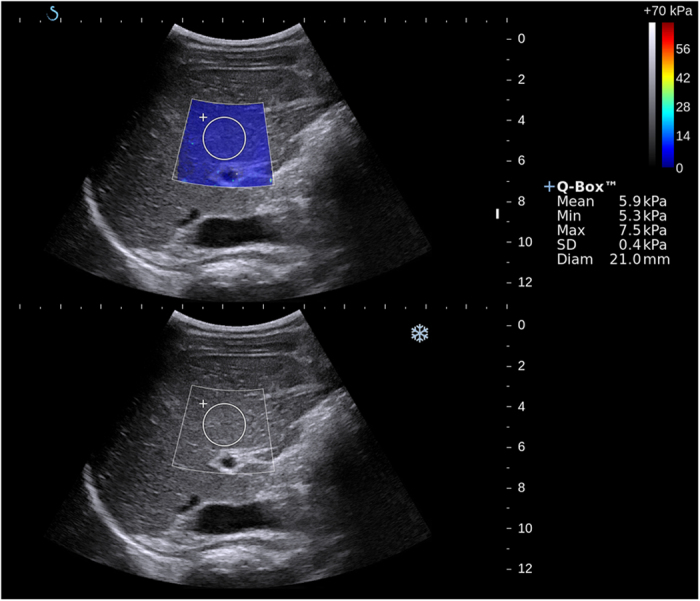
SSWE measurements in a 14-year old boy after KPE surgery. US images show a stiffness color map (top), which is homogeneous, with blue areas that correspond with low values of liver stiffness on the color scale, and SSWE measurements in the regions of interest (bottom) of mean 5.9 kPa.

SSWE was performed in dual mode (ie, elastograms displayed alongside gray-scale sonograms in real time). The operator chose the best static SSWE display images onto which a rectangular electronic region of interest (2.0 cm × 2.5 cm) and a circular region of interest (placed within the center of the rectangular region of interest, with diameter range from 1.5 cm to 2.5 cm) were positioned within 1.0 cm−3.0 cm from the capsular surface of the liver for analysis (Fig. 1). Once the optimal sizes of the regions of interest were chosen, they were fixed for subsequent measurements in each subject. Special attention was paid to avoid any focal lesions, vessels, biliary tracts, or artifacts from nearby lung gas. A successful SSWE was defined as the ROI box could be filled with color over 90% of the box. From the circular region of interest, the mean, standard deviation, and minimum and maximum kilopascal values were recorded. The average values from the three readings in each infant were used for subsequent statistical analyses.

#### Liver Histopathology

Under the guidance of US, liver biopsy was performed percutaneously in the right liver lobe using an inter-costal approach with an 18-gauge needle (Bard, USA). A minimum length of 20 mm for biopsy specimens must be guaranteed. Then, all specimens were fixed in formalin and embedded in paraffin. Following staining with haematoxylin-eosin (H&E) and Masson trichrome, each specimen was evaluated by two independent and blinded pathologists with more than 10 years of experience in liver pathology. Only the adequate samples defined by showing at least six portal tracts were included for further analysis^32^. Disagreement was resolved by the consultation of a third pathologist. Liver fibrosis and necroinflammatory activity was assessed using the Metavir classification as follows^31^,^33^: F0, no fibrosis; F1, portal fibrosis with no septa; F2, portal fibrosis with rare fibrous septa; F3, bridging fibrosis with many fibrous septa; F4,cirrhosis. Activity was staged as follows: A0, none; A1, mild; A2, moderate; A3, severe.

### Statistical Analysis

The normal distribution test was conducted in the variables using Shapiro-Wilk test.

Normally distributed variables were represented as mean ± SD while non-normally distributed variables such as skew variables were represented as median and inter-quartile range (IQR). Differences between two groups were compared by the t test for normally distributed variables, Wilcoxon rank test for skewed variables and χ2 test for categorical variables. Spearman’s rank coefficient test was performed to evaluate the correlation between variables. The Kruskal–Wallis test was used to detect the variation of SSWE values and APRI among different fibrosis stages. The diagnostic performance of SSWE, APRI and their combination to predict liver fibrosis severity (F0–1 vs. F2–4: F ≥ 2; F0-2 vs. F3–4: F ≥ 3; F0–3 vs. F4: F = 4) was estimated by the area under the receiver operating characteristic (ROC) curve. The optimal cut-off values were determined based on the largest Youden index. And the differences of AUROCs between different parameters were presented as P values estimated by Z tests. Statistical significance was considered as a two-sided P value of less than 0.05. All the analyses was performed by the SPSS 20.0 (SPSS Inc., Chicago, IL,USA), SAS 9.2 (SAS Institute, Inc, Cary, NC) and Sigmaplot 10.0. Ink (Systat Software, Inc.).

## Results

### Subject Characteristics

A total of 24 patients met the inclusion criteria for our study. Biochemical tests, SSWE examinations and liver biopsy were performed within one week of each other. In terms of fibrosis stage, there were 3 (12.5%) patients for F0, 2(8.3%) for F1, 4 (16.7%) for F2, 7 (29.2%) for F3, 8 (33.3%) for F4. The demographics, biochemical results, ultrasonic findings, histological features and follow-up data of patients by fibrosis level are summarized in Table 1. The mean age at liver biopsy was 6.6 years with 13 (54.2%) male patients. Patients with more advanced fibrosis stage were found to have significantly higher serum AST levels, GGT, DBIL levels and lower serum ALB level (*P* = 0.046; *P* = 0.049; *P* = 0.025; *P* = 0.044). SSWE values ranged from 6.2 to 60.6 (median, 13.2 kPa). On serum fibrosis biomarkers, the median value of APRI and FIB-4 was 1.3 (0.34–12.5) and 0.31(0.03–3.5) with the corresponding wide ranges. During the follow up from liver biospy, no liver-related death or gastrointestinal bleeding was detected. There were two patients in need of liver transplantation (one with F4 has underwent surgery and one with F2 is on the waiting list). Three patients showed with ascites(1 with F4, 1 with F3 and 1 with F2).

### Performance of APRI, FIB-4 and SSWE in Predicting Liver Fibrosis Severity

**Figure 2:**
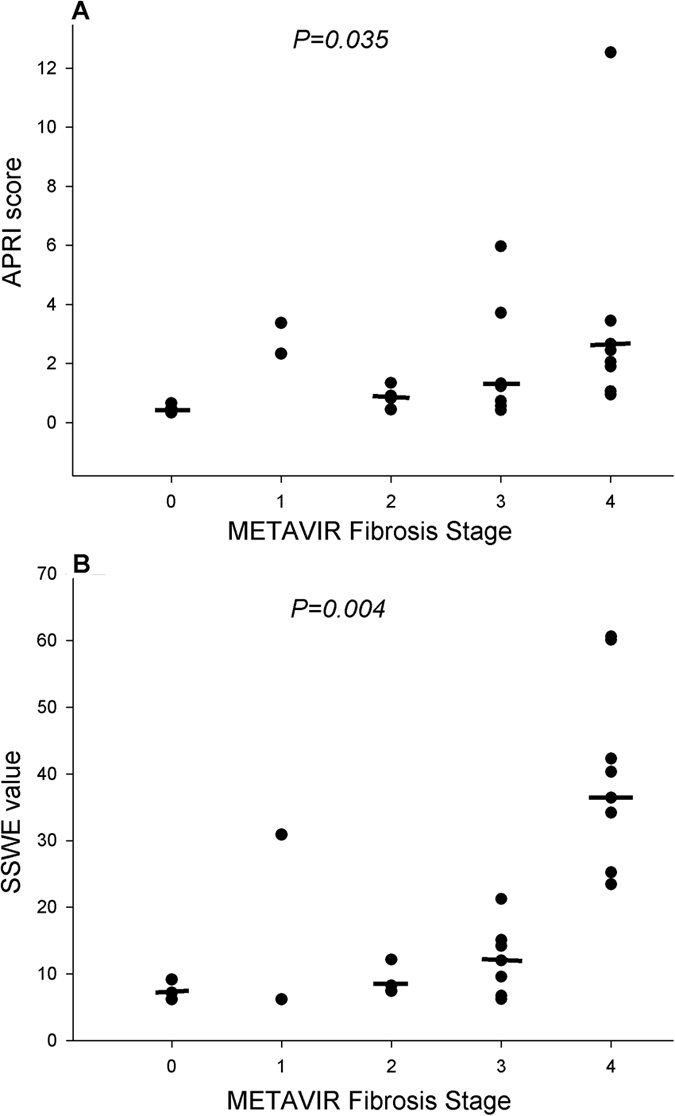
APRI scores (**A**) and SSWE values (**B**) for each Metavir fibrosis stage. The line across the dots indicate the median value.

**Figure 3:**
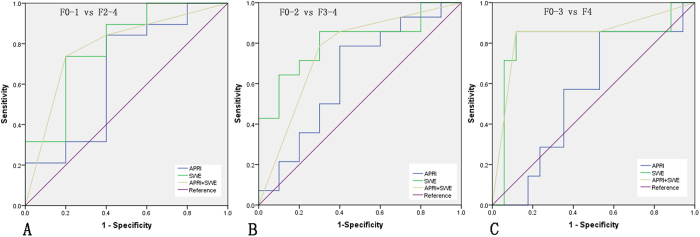
ROC curves for SSWE, APRI and their combination in predicting different fibrosis thresholds: F2-4 vs. F0-1 (**A**), F3-4 vs. F0-2 (**B**) and F4 vs. F0-3 (**C**).

APRI scores had a positive correlation with fibrosis stage (r = 0.583, *P* < 0.001) (Supplementary Figure A) whereas FIB-4 scores had a very weak correlation (r = 0.075, *P* = 0.001). APRI scores showed a significant difference between different fibrosis stages (*P* = 0.035; Fig. 2A) whereas FIB-4 scores did not. Thus, we moved to further explore the performance of APRI in predicting fibrosis severity. A significant difference was detected between consecutive fibrosis stages except F3 and F4 (F0 vs. F1: *P* < 0.001; F1 vs. F2: *P* < 0.001; F2 vs. F3: *P* = 0.009; F3 vs. F4: *P* = 0.323). Moreover, the AUROCs of APRI were 0.65 (95% CI: 0.35–0.96), 0.64 (95% CI: 0.41–0.88), and 0.56 (95% CI: 0.31–0.80) respectively for predicting significant (≥F2), advanced fibrosis stage (≥F3), and cirrhosis (F4) (Table 2, Fig. 3). With the corresponding cut-off values of 0.70, 0.93 and 1.00 in the above three situations, the performance of APRI in predicting fibrosis severity was estimated (Table 2). SSWE values showed a positive correlation with fibrosis stage (r = 0.762, *P* < 0.001) (Supplementary Figure B) and exhibited significant difference among different fibrosis stages (*P* = 0.004; Fig. 2B). There was significant difference between consecutive fibrosis stages except F2 and F3 (F0 vs. F1: *P* < 0.001; F1 vs. F2: *P* < 0.001; F2 vs. F3: *P* = 0.450; F3 vs. F4: *P* = 0.001). Compared to APRI, SSWE had larger AUROCs in predicting significant fibrosis (0.79, 95% CI: 0.54–1.00), advanced fibrosis (0.81, 95% CI: 0.63–0.99) and cirrhosis (0.82, 95% CI: 0.58–1.00) with the corresponding cut-off values of 9.4, 10.8 and 24.4 kPa (Table 2, Fig. 3). However, the difference between SSWE and APRI in predicting significant fibrosis was not significant (*P* = 0.65) whereas the differences in predicting advanced fibrosis and cirrhosis were statistically significant (both *P* < 0.001). The details on the diagnostic accuracy of SSWE are presented in Table 2.

Moreover, SSWE demonstrated a positive correlation with APRI with significant difference (r = 0.543, *P* = 0.006). Combining SSWE with APRI did not improve the predictive power in significant or advanced fibrosis over SSWE alone, with the AUROC being 0.78 (95% CI: 0.55–1.00) for significant fibrosis and 0.76 (95% CI: 0.55–0.97) for advanced fibrosis, respectively. However, the combination of SSWE and APRI could slightly improve the diagnostic accuracy for cirrhosis, with the AUROC being 0.84 (95% CI: 0.63–1.00) (Table 2). However, the difference between combination and SSWE was not statistically significant (*P* = 0.33).

### Discordance between SSWE, APRI and Fibrosis Stage

Based on the above cut-off values, the predicted fibrosis stage by SSWE and APRI and the actual fibrosis level was compared. APRI was in agreement with fibrosis staging 50% (12/24) of the time. Overall, APRI overestimated fibrosis stage 29.2% of the time and underestimated fibrosis 20.8% of the time. For SSWE, more patients (15, 62.5%) were diagnosed with agreement to actual fibrosis stage than APRI. SSWE overestimated fibrosis stage in 12.5% of study subjects and underestimated fibrosis in 25.0% of study subjects. More specifically, about 25.0% (6 of 24) of the patients had discordance of one stage between SSWE value and Metavir score. About 12.5% (3 of 24) of the patients had discordance of two stages or more between SSWE value and Metavir score. For the six underestimated cases by SSWE, there were two patients with SSWE values less than 7.0 but turned out to be in F3 stage by histology. These patients have low APRI, normal PLT count, normal serum bilirubin level and albumin level.

There were two patients with SSWE values of >10.8 kPa but without advanced fibrosis or cirrhosis on liver biopsy. To look further into these patients, we found that they had other biochemical and ultrasonic manifestations suggesting of advanced fibrosis or cirrhosis, such as APRI ≥ 1.0, low PLT count, high serum AST level and splenomegaly (Table 3).

### Correlation between SSWE and Liver Function Biomarkers after 6 months from biopsy

In order to preliminarily investigate the role of SSWE in predicting patients’ liver function in future day, we analyzed the correlation between our SSWE results and liver function biomarkers after 6 months from liver biopsy. It showed that SSWE had significant negative correlation with PLT count (r = −0.426, *P* = 0.038) and serum ALB level (r = −0.670, *P* < 0.001). Besides, SSWE values appeared to be positively correlated with serum TBIL (r = 0.419, *P* = 0.041) and DBIL levels (r = 0.518, *P* = 0.010) at 6 months after liver biopsy.

## Discussion

Our study revealed that SSWE examination had a promising diagnostic accuracy to predict liver fibrosis stage, which is better than APRI. SSWE values had significant positive correlations with liver fibrosis severity in patients after kasai surgery. Moreover, SSWE values were correlated with biological parameters of liver function at 6 months after liver biopsy.

Liver stiffness measurement has become a reliable tool to assess liver fibrosis in adult patients with chronic liver diseases^19^,^20^,^21^,^34^,^35^, however, the evaluation of its use in children is limited. Until now, there are a few studies on assessing the predictive power of Fibroscan, TE, ARFI in fibrosis stage for children with various liver diseases^7^,^8^,^9^. To the best of our knowledge, this is the first study to investigate the performance of SSWE in predicting liver fibrosis for postoperative children with BA and to compare with those of APRI and FIB-4. The reported cutoff values of TE for predicting advanced fibrosis and cirrhosis in previous literatures varied, ranging from 7.9 to 11 and 11.0 to 25.8 kPa, respectively^36^,^37^,^38^,^39^. Besides, a study on the performance of SSWE in the evaluation of liver fibrosis for children reported a cut-off value of 10.4 kPa for predicting significant fibrosis^15^. In our study, the cut-off values for diagnosis of significant fibrosis, advanced fibrosis and cirrhosis were 9.4, 10.8 and 24.4 kPa, which were within the ranges presented in the previous studies. However, we should note that the cutoff values for diagnosing liver fibrosis differ depending on the method we used. Compared to the results of Fibroscan in previous pediatric BA studies (AUROC of 0.87–0.88 for F = 4)^26^,^40^, SSWE yielded similar performance characteristics with AUROC of 0.82 in our study. Shin NY *et al*. reported the AUROCs of 0.86 and 0.96 respectively in predicting severe fibrosis and cirrhosis for TE in 47 infants with BA^7^, which was slightly better than those of our study. This might be probably due to the relatively poorer discriminative power of our small sample size. For ARFI, Shima H *et al*. only suggested a correlation between ARFI and fibrosis stage in 8 patients with BA and there was no data regarding AUROC of ARFI in predicting fibrosis in BA patients^8^. Since there was limited data on the efficacy of SSWE in liver fibrosis for pediatric patients, we tried to compare them with those of adult studies. Two adult studies reported the AUROCs of 0.88 to 0.90 and 0.90 to 0.93 for SSWE in predicting significant fibrosis and severe fibrosis, respectively^41^,^42^. Compared to these, the diagnostic accuracy of SSWE was slightly poorer in our study. The underlying explanations might be as follows. First, adults could corporate better than children with complete breath holding during the SSWE examination and the influence of respiration to SSWE results could not be completely reduced by sedation for pediatric patients. Second, the serum levels of bilirubin especially the DBIL could affect the SSWE values, and they were relatively not high for patients in adult studies but might be abnormally high for some children in our study. It was reported that bile duct obstruction with increased level of bilirubin could influence the evaluation of liver stiffness by over-diagnosis of liver cirrhosis^43^. The mechanisms behind the high stiffness in cholestasis remained unclear but might be probably associated with tissue swelling, inflammation, edema, and increased intracellular pressure^43^. Therefore, the threshold values of SSWE for different fibrosis stages were relatively higher in our study than those in adult studies. Third, the age range of our patients might be a little wide resulting in poor homogeneity of the study subjects. However, it was reported that there was no significant difference in SSWE values among different age groups^22^ which meant that age might not influence the SSWE results to a dominant level. Fourth, it remained uncertain whether there existed differences between children and adults, or between BA and other kinds of liver diseases for the performance of SSWE in predicting fibrosis. This required further studies to elucidate. In addition to its promising efficacy in predicting fibrosis, SSWE has technical and operating advantages over TE and ARFI. Unlike TE, SSWE can be performed with conventional US probes during an abdominal ultrasound scan as it was integrated into a conventional US system, which was much more convenient. Moreover, SSWE can locate ROI precisely with avoiding large vessels and bile ducts and real-time quantitative analysis of liver stiffness. Thus, SSWE, as a non-invasive, convenient, readily available and non-expensive technique, has shown encouraging predictive accuracy in liver fibrosis, which was critical for identifying high-risk patients and disease monitoring. This importance was especially significant for BA children after KPE who required close surveillance and hence were with more demanding on non-invasive tests due to frequent reexaminations.

APRI and FIB-4, as the noninvasive, readily available biomarkers, have performed well in predicting fibrosis in various adult liver diseases with AUROCs of more than 0.80^23^,^24^,^25^. Previous studies were consistent in reporting that APRI was superior to FIB-4 in predicting fibrosis stage for pediatric liver diseases^28^,^44^. As in our study, FIB-4 completely failed to be the reliable fibrosis biomarker (AUROC < 0.50). However, controversy remained regarding the diagnostic accuracy of APRI in liver fibrosis for children. On one hand, Leung DH *et al*. reported that APRI exhibited a high AUROC (0.81) in predicting advanced liver fibrosis in children with cystic fibrosis liver disease^44^. Kim SY *et al*. also suggested that APRI was a promising surrogate of advanced fibrosis (AUROC = 0.92) and cirrhosis (AUROC = 0.91) in children with BA^45^. On the other hand, a study from China reported a suboptimal performance of APRI in predicting cirrhosis (AUROC = 0.54) for children with BA^40^. A multicenter data from U.S.A also demonstrated that APRI had poor diagnostic accuracy for significant (AUROC = 0.67) or advanced fibrosis (AUROC = 0.63) in children with nonalcoholic fatty liver disease^28^. In our study, APRI proved to be a poor predictor for liver fibrosis in children with BA (AUROC = 0.65, 0.64, 0.56 for F ≥ 2, F ≥ 3, F = 4, respectively), which was consistent with the above two studies. Therefore, although APRI show extremely promising results in adult studies, the results could not be confirmed in BA children after KPE based on the data in this and previous studies. Besides, SSWE outperformed APRI in predicting liver fibrosis in our study. APRI did not seem to improve the diagnostic accuracy and reliability of SSWE in evaluating fibrosis stages. This might be explained by the fact that SSWE could reflect fibrosis severity with application of local mechanical compression on liver tissue using focused ultrasonography and acquiring strain images whereas the parameters used in the APRI such as AST and ALT are increased because of cholestasis itself or liver disease, in which their power in evaluating fibrosis stages is very limited. Since SSWE could reflect a patient’s liver fibrosis stage to some extent whereas laboratory tests could not, SSWE has the advantage over them as the tool to predict prognosis and to assist with designing future treatment plan. Moreover, SSWE is cheaper than laboratory tests. In such a limited resource world, SSWE may be cost-effective.

Furthermore, the effective measurement of liver fibrosis may help predict the liver function during follow-up. Thus, we turned to investigate the correlation of SSWE with the biochemical parameters of liver function at 6 months after SSWE. Results showed that SSWE were negatively correlated with PLT and ALB which reflected the extent of portal hypertension and synthesis function of liver. SSWE appeared to be moderately correlated with bilirubin (TBIL or DBIL) which was the recognized factor associated with prognosis of patients with BA. Thus, these results showed that SSWE might probably serve as the predictor of the prognosis of liver fibrosis during follow-up, which was consistent with the previous study suggesting that liver stiffness measurement values of 3 months after KPE can be used to predict the development of liver related events^46^. However, future studies with large sample size are needed to validate the above results.

In our study, there was two patients’ fibrosis stages over-diagnosed by SSWE. However, careful analysis of these cases suggested that they both had laboratory features suggestive of portal hypertension or actual cirrhosis. This indicates that the gold standard of liver biopsy is not always reliable due to the sampling error and the subjective nature of the reading. We should evaluate a patient’s fibrosis stage based on pathologic results together with clinical, laboratory and ultrasonic features.

There are a number of limitations to our study. First, the sample size is small without even distribution of patients in different fibrosis stage. The cut-off value chosen here is a relatively limited result based on the data of 24 patients. These factors make the statistical power of this study low. Second, it is a retrospective study with all its inherent defects. Third, our study excluded the patients with failure in performing SSWE examinations, in which we could not investigate the applicability of SSWE examinations in children with BA. In conclusion, SSWE might be a reliable and noninvasive tool for assessing liver fibrosis in postoperative children with BA whereas APRI showed poor predictability for fibrosis stage. However, due to the relatively low statistical power of this study, the findings here need to be validated in large prospective studies with long follow-up time in the future.

## Additional Information

**How to cite this article**: Chen, S. *et al*. Supersonic shearwave elastography in the assessment of liver fibrosis for postoperative patients with biliary atresia. *Sci. Rep*. **6**, 31057; doi: 10.1038/srep31057 (2016).

## Supplementary Material

Supplementary Information

## Figures and Tables

**Table 1:** Patient demographics and clinical characteristics.

Variable	Total	F0-2	F3-4	*P* value
Sex, n (%)	24	9	15	0.341
Men	13	6	7	
Female	11	3	8	
Age at KPE, months (SD; range)	2.1 (1.1; 0.50–6.0)	2.5 (1.5; 1.0–6.0)	1.8 (0.6; 0.50–3.0)	0.124
Follow-up time, months (SD; range)	32.8 (7.6; 6.0–39.0)	35.9 (4.8; 27.0–39.0)	31.0 (8.5; 6.0–39.0)	0.131
*During the liver biopsy period*				
Age, years (SD; range)	6.6 (5.7; 0.67–20.0)	8.4 (5.1; 1.5–16.0)	5.5 (5.9; 0.67–20.0)	0.089
AST, IU/L (IQR; range)	62.0 (40.0–120.0; 27.0–533.0)	40.0 (33.5–82.5; 27.0–136.0)	83.0 (48.0–128.0; 27.0–533.0)	0.046
ALT, IU/L (IQR; range)	53.5 (27.3–80.8; 14.0–558.0)	42.0 (21.0–73.5; 14.0–136.0)	68.0 (31.0–91.0; 14.0–558.0)	0.340
ALP, IU/L (IQR; range)	274.0 (177.5–455.3; 83.0–703.0)	253.0 (145.0–309.0; 83.0–335.0)	289.0 (209.0–530.0; 106.0–703.0)	0.089
GGT, IU/L (IQR; range)	167.5 (66.3–250.8; 14.0–468.0)	89.0 (39.0–177.5; 14.0–251.0)	240.0 (91.0–325.0; 39.0–468.0)	0.049
ALB, g/L (SD; range)	43.3 (4.3; 35.9–51.8)	45.5 (3.5; 38.6–51.8)	42.0 (4.7; 35.9–47.8)	0.044
TBIL, μM/L (IQR; range)	10.7 (6.9–16.8; 3.9–138.2)	7.5 (6.2–11.3; 4.1–13.2)	13.3 (7.0–20.8; 3.9–138.2)	0.107
DBIL, μM/L (IQR; range)	3.2 (2.1–8.7; 0.7–69.8)	2.7 (1.2–3.4; 0.7–4.1)	7.2 (2.4–11.6; 0.90–69.8)	0.025
PLT; 10^9^/L (IQR; range)	148.5 (107.5–181.0; 40.0–308.0)	151.0 (115.5–204.5; 91.0–234.0)	146.0 (82.0–182.0; 40.0–308.0)	0.676
APRI (IQR; range)	1.3 (0.68–2.6; 0.34–12.5)	0.82 (0.46–1.84; 0.34–3.4)	1.9 (0.95–3.5; 0.42–12.5)	0.053
FIB-4 (IQR; range)	0.31 (0.13–0.51; 0.03–3.5)	0.40 (0.19–0.45; 0.14–0.98)	0.22 (0.08–0.54; 0.03–3.54)	0.493
SSWE, kPa (IQR; range)	13.2 (7.4–33.4; 6.2–60.6)	7.5 (6.7–10.7; 6.2–30.9)	23.5 (12.0–40.3; 6.2–60.6)	0.008
*Follow-up*				
Prolonged jaundice	2	1	1	0.703
Ascites	3	1	2	0.692
Gastrointestinal bleeding	0	0	0	1.000
Hepatic encephalopathy	0	0	0	1.000
Liver transplantation	2	1	1	0.703
Liver related death	0	0	0	1.000

KPE, Kasai portoenterostomy; SD, standard deviation; IQR, inter-quartile range; AST, aspartate aminotransferase; ALT, alanine amino-transferase; ALP, alkaline phosphatase; GGT, gamma-glutamyl transpeptidase; ALB, albumin; TBIL, total bilirubin; DBIL, direct bilirubin; PLT, platelet; APRI, aspartate aminotransferase to platelet ratio index; FIB-4, fibrosis-4; SSWE, supersonic shearwave elastography.

**Table 2:** Diagnostic accuracy of APRI and SSWE for liver fibrosis based on optimal cut-off values in children with BA.

	F2-4 vs. F0-1	F3-4 vs. F0-2	F4 vs. F0-3
*APRI*			
Cut-off	0.70	0.93	1.00
AUROC (95% CI)	0.65 (0.35–0.96)	0.64 (0.41–0.88)	0.56 (0.31–0.80)
Sensitivity, % (95% CI)	60.0 (17.0–92.7)	66.7 (30.9–91.0)	56.3 (30.6–79.2)
Specificity, % (95% CI)	84.2 (59.5–95.8)	80.0 (51.4–94.7)	87.5 (46.7–99.3)
PPV, % (95% CI)	50.0 (13.9–86.1)	66.7 (30.9–91.0)	90.0 (54.1–99.5)
NPV, % (95% CI)	88.9 (63.9–98.1)	80.0 (51.4–94.7)	50.0 (24.0–76.0)
*SSWE*			
Cut-off	9.4	10.8	24.4
AUROC (95% CI)	0.79 (0.54–1.00)	0.81(0.63–0.99)	0.82 (0.58–1.00)
Sensitivity, % (95% CI)	80.0 (29.9–98.9)	77.8 (40.2–96.1)	93.8 (67.7–99.7)
Specificity, % (95% CI)	73.7 (48.6–89.9)	80.0 (51.4–94.7)	87.5 (46.7–99.3)
PPV, % (95% CI)	44.4 (15.3–77.3)	70.0 (35.4–91.9)	93.8 (67.7–99.7)
NPV, % (95% CI)	93.3 (66.0–99.7)	85.7 (56.2–97.5)	87.5 (46.7–99.3)
*APRI* + *SSWE*			
AUROC (95% CI)	0.78 (0.55–1.00)	0.76 (0.55–0.97)	0.84 (0.63–1.00)
Sensitivity, % (95% CI)	60.0 (17.0–92.7)	44.4 (15.3–77.3)	50.0 (25.5–74.5)
Specificity, % (95% CI)	73.7 (48.6–89.9)	86.7 (58.4–97.7)	87.5 (46.7–99.3)
PPV, % (95% CI)	37.5 (10.2–74.1)	66.7 (24.1–94.0)	88.9 (50.1–99.4)
NPV, % (95% CI)	87.5 (60.4–97.8)	72.2 (46.4–89.3)	46.7 (22.3–72.6)

APRI, aspartate aminotransferase to platelet ratio index; SSWE, supersonic shear wave elastography; BA, biliary atresia; AUROC, area under the receiver operating characteristic curve; CI, confidence interval; PPV, positive predictive value; NPV, negative predictive value.

**Table 3:** Clinical manifestations of patients with high SSWE values (>10.8 kPa) but without advanced fibrosis or cirrhosis in liver biopsy.

Patient	Fibrosis stage	SSWE, kPa	APRI	FIB-4	PLT, 10^9^/L	AST, IU/L	ALB, g/L	TBIL, μM/L	Spleen, cm	Ascites	LTx
1	1	30.9	2.33	0.14	122	105	38.6	6.3	12.7	No	No
2	2	12.2	1.34	0.23	91	45	41	7.2	13.5	No	No

SSWE, supersonic shear wave elastography; BA, biliary atresia; APRI, aspartate aminotransferase to platelet ratio index; FIB-4, fibrosis-4; PLT, platelet; AST, aspartate aminotransferase; ALB, albumin; TBIL, total bilirubin; LTx, liver transplantation.
