# Supplementary material for: Supersonic shearwave elastography in the assessment of liver fibrosis for postoperative patients with biliary atresia
Source: Sci Rep. 2016 Aug 11;6:31057. doi: 10.1038/srep31057 (PMC4980634; doi:10.1038/srep31057)
Supplement: Supplementary Information [file srep31057-s1.pdf]

Supersonic shearwave elastography in the assessment of liver fibrosis for postoperative patients with biliary atresia

Shuling Chen, M.D.[1#], Bing Liao, M.D., Ph.D.[2#], Zhihai Zhong, M.D.[3], Yanling Zheng, M.D.,Ph.D[1], Baoxian Liu, M.D.[1], Quanyuan Shan, M.D.[1],Xiaoyan Xie, M.D., Ph.D.[1*], Luyao Zhou, M.D.[1*]

Supplementary Table. Patients' ultrasonic features and pathological data

Variable	Total	F0-2	F3-4	*P* value
**Liver size, cm (SD; range)**	10.9(1.3; 8.4-14.7)	10.7(1.1; 8.4-12.1)	11.0(1.5; 8.9-14.7)	0.670
**Spleen size, cm (SD; range)**	11.7(2.1; 8.1-16.4)	12.0(1.7; 9.7-14.6)	11.5(2.3; 8.1-16.4)	0.591
**Portal vein diameter, cm (IQR; range)**	0.75(0.40-3.6;0.60-1.0)	0.80(0.70-1.1; 0.50-1.2)	0.60(0.60-1.0; 0.40-3.60)	0.228
**PVV, cm/s (IQR; range)**	21.1(17.7-25.9; 12.5-58.4)	18.7(17.7-22.0; 16.1-25.9)	25.2(17.5-28.8; 12.5-58.4)	0.114
**Inflammatory activity grade**				0.062
**A0**	2	1	1	
**A1**	11	7	4	
**A2**	7	1	6	
**A3**	4	0	4	
**Ductual proliferation**				0.048
**0**	10	7	3	
**1**	6	1	5	
**2**	6	1	5	
**3**	2	0	2	

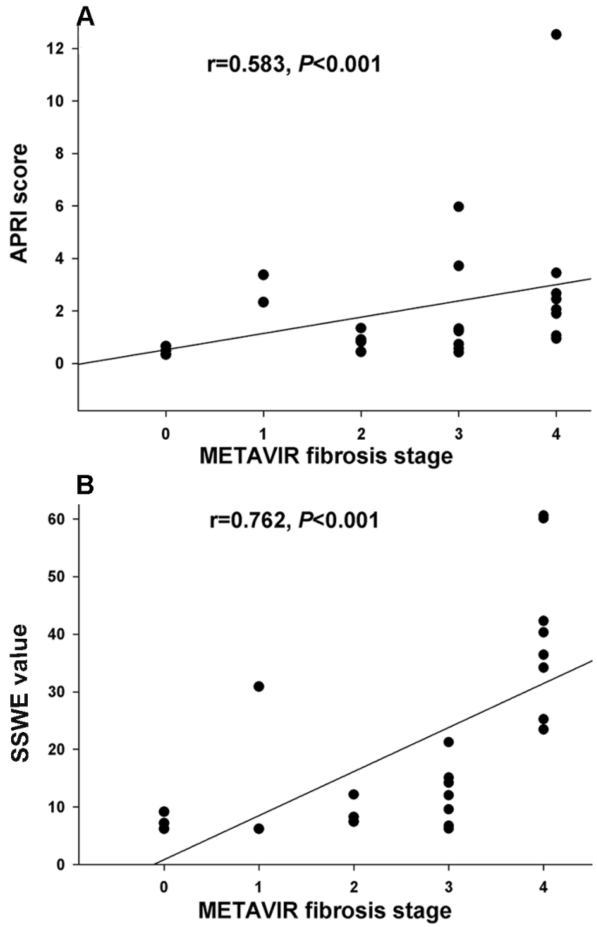

Supplementary Figure. Correlations between APRI score (A), SSWE value (B) and Metavir fibrosis stage.
